# Prognosis of sepsis induced by cecal ligation and puncture in mice improved by anti-*Clonorchis Sinensis* cyclopholin a antibodies

**DOI:** 10.1186/s13071-015-1111-z

**Published:** 2015-10-01

**Authors:** Tianzhang Song, Mei Yang, Jintao Chen, Lilin Huang, Hongling Yin, Tailong He, Huaiqiu Huang, Xuchu Hu

**Affiliations:** Department of Parasitology, Zhongshan School of Medicine, Sun Yat-sen University, Guangzhou, Guangdong China; Education Ministry Key Laboratory for Tropical Disease Control Research, Sun Yat-sen University, Guangzhou, Guangdong China; Department of Dermatology and Venereology, The Third Affiliated Hospital of Sun Yat-sen University, Guangzhou, Guangdong China

**Keywords:** *Clonorchis sinensis*, Cyclophilin A, Sepsis

## Abstract

**Background:**

Cyclophilin A (CyPA), a ubiquitously distributed intracellular protein, is thought to be one of the important inflammatory factors and plays a significant role in the development process of sepsis. In the form of cytokine, CyPA deteriorates sepsis by promoting intercellular communication, apoptosis of endothelial cells and chemotactic effect on inflammatory cells. In our previous study, cyclophilin A of *Clonorchis sinensis* (CsCyPA), a type of excretory-secretory antigen, could induce the patients infected with *Clonorchis sinensis* to produce specific anti-CsCyPA antibodies. In this study, we investigated whether anti-CsCyPA antibodies could cross-react with CyPA and then play a protective role against sepsis, just like other anti-cytokine antagonists.

**Methods:**

The mice model with sepsis was established with cecal ligation and puncture (CLP). Fifty mg/kg purified anti-CsCyPA antibodies were injected via the caudal vein 6 h after the CLP operation, and persistent observation was performed for 72 h. Blood samples and tissues were collected at 6 h, 12 h, 24 h, 48 h and 72 h after CLP. Cytokines in serum were measured by ELISA. Lung and mesentery tissues were stained with hematoxylin-eosin. Endothelial cells (ECs) isolated from murine aorta were co-cultured with CyPA of mice (MuCyPA) and anti-CsCyPAs for 24 h, then, viability was measured by Cell Counting Kit-8.

**Results:**

Anti-CsCyPA antibodies could combine with MuCyPA and inhibite its peptidyl prolyl isomerase (PPIase) activity. In the antibodies treatment group, blood coagulation indicators including PT, aPTT, D-dimer and platelet count were obviously more ameliorative, the proinflammary factors like IL-6, TNF-α, IL-1β were significantly lower at 12 h and 24 h after surgery and the viability of ECs was significantly improved compared to those in the control group. Furthermore, the survival rate was elevated, ranging from 10.0 % to 45.0 % compared to the control group.

**Conclusions:**

These antibodies may have a favorable effect on sepsis via inhibition of enzymic activity or protection of endothelial cells.

## Background

Sepsis is defined as a systemic inflammatory response syndrome (SIRS) coupled with a documented infection that may result in septic shock and multiple organ failure (MOF) [1]. The sepsis mortality in humans has been high at more than 50 % [2]. SIRS serves as a hallmark sign of sepsis, and is characterized by a hyperinflammatory response of the host to invading pathogens that are primarily mediated by cytokines [3]. However, treatment of patients suffering from sepsis with traditional proinflammatory cytokine antagonist, such as anti-TNF-α, interleukin-1 receptor antagonist, bradykinin antagonist and others, did not prove effective in controlling multi-organ damage and mortality [4].

Cyclophilins (CyPs) are a family of ubiquitous proteins evolutionarily well conserved and present in all prokaryotes and eukaryotes [5]. Equipped with PPIase activity, CyPs catalyze the isomerization of peptide bonds from the *trans* form to *cis* form at proline residues and facilitate protein folding [6]. Cyclophilin A (CyPA), a universally expressed protein belonging to the CyPs family, can be secreted from cells in response to inflammatory stimuli such as hypoxia, infection, sepsis and oxidative stress [7–10]. In the form of cytokine, CyPA deteriorates sepsis by promoting intercellular communication, apoptosis of endothelial cells and chemotactic effect on inflammatory cells [11].

*Clonorchis sinensis* (*C. sinensis*), causing clonorchiasis, has been one of the most important food-borne parasites in China [12]. Most people infected with *C.sinensis* present no apparent clinical manifestations. Only 5 %–10 % of infected people have non-specific symptoms such as abdominal pain in the right upper quadrant, flatulence, and fatigue [13, 14]. A *C.sinensis* adult full-length complementary DNA (cDNA) plasmid library was established in our laboratory in 2004 [15]. CsCyPA was found to be an excretory protein and able to induce high anti-CsCyPA antibodies (anti-CsCyPAs) titers in patients infected with *C.sinensis* in our previous study [16].

In 1989, David P Strachan proposed a hygiene hypothesis, according to which the decreased incidence of infections with parasites in developed countries may be the underlying cause for some diseases [17, 18]. Nowadays, parasites and their products constitute the targets of studies as a potential alternative approach for parasitic, viral, bacterial, and autoimmune diseases [19–21]. Therefore, the aim of this study was to determine whether anti-CsCyPAs could, like other anti-cytokine antagonists, play a protective role against sepsis.

## Methods

### Preparation of recombinant CyPA and polyclonal antibodies

Recombinant CsCyPA (rCsCyPA) was produced in a previous study [16]. Furthermore, recombinant CyPA of *Schistosoma japonicum* (rSjCyPA), mouse (rMuCyPA) and human (rHsCyPA) were produced using the same process.

Six SD rats were divided randomly into two groups, one group was injected subcutaneously with 100 μg rCsCyPA emulsified with equal volume of complete Freund’s adjuvant (CFA, Sigma), followed by three boosts with 50 μg antigen emulsified with incomplete Freund’s adjuvant (IFA, Sigma) at 2-week intervals. The other group was immunized with PBS as control. Two weeks after the last vaccination, serum samples were collected from the mice and the rCsCyPA-specific IgG detected by ELISA.

Antisera were precipitated three times with ammonium sulphate (33 % saturation), the pellet dissolved in TBS buffer (20 mM Tris–HCl, pH 7.5, 0.15 M NaCl) and dialyzed against the same buffer for 18 h. Antibodies were purified by affinity chromatography on a G-Sepharose column. Antibodies were eluted from the column with 0.1 M glycine-HCl, pH8.8, and then, dialyzed against TBS solution for 18 h. The concentration of anti-CsCyPAs was measured by using a BCA Protein Assay Kit (Thermo, USA) following the manufacturer’s instructions.

### Identification by Western blot analysis

The purified rCsCyPAs, rSjCyPA, rMuCyPA and rHsCyPA (25 ug of each protein) were subjected to SDS-PAGE (12 %). After electrotransferral to a polyvinylidene difluoride (PVDF) membrane (Whatman), the blotted membranes were probed with anti-CsCyPAs or PBS and subsequently incubated with horseradish peroxidase (HRP)-conjugated goat anti-rat secondary antibody (Santa Cruz). Finally, the result was visualized using diaminobenziine (DAB, Boster, Wuhan, China) substrate solution.

### PPIase activity and inhibition

Colorimetric detection of PPIase activity was performed by the chymotrypsin-coupledcleavage assay according to Fischer et al. [22]. Briefly, 10 ug of rMuCyPA per reaction system was co-cultured with 1 ug or 10 ug anti-CsCyPAs for 1 h at 37 °C before experiment. The enzymatic activity was performed in 50 mM HEPES (N-2-hydroxyethylpiperazine-N’-2-ethanesulfonic acid) buffer, pH 8.0, at 10 °C. The reaction was started by the addition of the synthetic peptide Suc-Ala-Phe-Pro-Phe-p-nitroanilide. P-nitroaniline chromophore release from the all-trans peptide was monitored at 390 nm using the Infinite F500 (TECAN, Swit).

### CLP model and anti-CsCyPAs treatment

#### Ethical approval

All animal experiments in this paper were performed in strict accordance with the Guide for the Care and Use of Laboratory Animals of Sun Yat-sen University (Permit Numbers: SCXK (Guangdong) 2009–0011). 220 KM male mice (5–6 weeks of age and weighing 20-22 g) were purchased from the Experimental Animal Center of Sun Yat-sen University (Guangzhou, China) housed in a temperature controlled, light-cycle room in animal facilities, with unlimited food and water.

Sepsis was induced in the mice model by CLP [23]. Twenty mice were selected randomly from the total animals as a sham-surgery group. The other two hundred were randomly divided to two groups: <1 > The sham group skipped the steps of cecal perforation, instead the peritoneum was immediately closed after exposure of the cecum. Normal saline (150 μl) was injected via the caudal vein 6 h after surgery. <2 > CLP control group were injected with 150 μl of normal saline via caudal vein 6 h after CLP surgery. < 3 > CLP treatment group were injected with 150 μl of normal saline including 50 mg/kg of purified antibodies. Mice in each group were divided equally into five subgroups, which were sacrificed at 6, 12, 24, 48 and 72 h respectively after surgery. There were four mice in each sham surgery subgroup, twenty in each CLP treatment subgroup and twenty in each CLP control group. In each subgroup, survival mice were anesthetized with diethyl ether, then blood collected into anticoagulant and coagulant tubes through the eyeball and the mesentery and lungs separated. The detection of survival rate was presented in the 72 h subgroup.

### Measured cytokine and CyPA in serum

Blood samples in each subgroup collected in coagulant tubes (B&D, USA) were clotted for two hours at room temperature before centrifugation for 15 min at 1000xg. Serum was removed and stored samples at −80 °C. MuCyPA were measured using a mouse cyclophilin A ELISA Kit (CUSABIO, USA). TNF-α, IL-6, IL-1β, IL-4, IL-10 and IFN-γ were determined by the corresponding ELISA Kit (R&D, USA) according to the manufacturer’s instructions. All samples were measured at OD_450nm_ in a Sunrise Absorbance reader (TECAN, Swit).

### Pathological observation of lung and mesentery tissues

The mesentery and lung tissues were fixed in 10 % formaldehyde for 24 h and then embedded in paraffin. Subsequently, the paraffin-embedded samples were cut into 5 μm thick sections and stained with hematoxylin-eosin. All samples were photographed and examined immediately by Leica DM Microscopes (DM 2500B, Germany, ×400).

### Blood coagulation indicator

Blood samples in each subgroup collected by anticoagulant tubes (Improve Medical, Guangzhou, China) were texted within four hours. Prothrombin time (PT), activated partial thromboplastin time (aPTT) and fibrinogen were detected in an automated coagulometer (Sysmex CS2000i; Fuji, Japan). Platelet count was performed using an automatic blood cell counter (Sysmex XS1000i; Fuji, Japan). D-dimer was detected by D2D ELISA Kit (R&D Systems, USA).

### Vascular endothelial cells isolation, culture and treatment

The isolation of ECs from murine aorta was described by Mika Kobayashi in 2005 [24]. Briefly, the aorta of KM mice was dissected out from the aortic arch to the abdominal aorta, and the connective tissues removed under a stereoscopic microscope. ECs were isolated from aorta by collagenase type II solution (2 mg/ml, dissolved in serum-free DMEM) for 45 min at 37 °C, then, seeded into 96-well plates at 5,000 cells per 100 μl for each well with endothelial cell phenol red free culture medium (Sciencell, USA).

Different concentrations of rMuCyPA and anti-CsCyPAs were co-cultured with ECs for 72 h before being measured by Cells Counting Kit-8 (CCK-8) (Beyotime, Jiangsu, China) according to the manufacturer’s instructions. Each well was incubated with 10 μl of CCK-8 solution at 37 °C for 2 h and measured at OD_450nm_ in a Sunrise Absorbance reader (TECAN, Swit). Each experiment was repeated three times.

### Statistical analysis

Date was reported as the mean ± SD. All statistical analysis was performed using Prism 5.0 (GraphPad Software, USA). A significance level of 0.05 was considered to be significant for all calculations.

## Results

### Homology analysis

**Fig 1:**
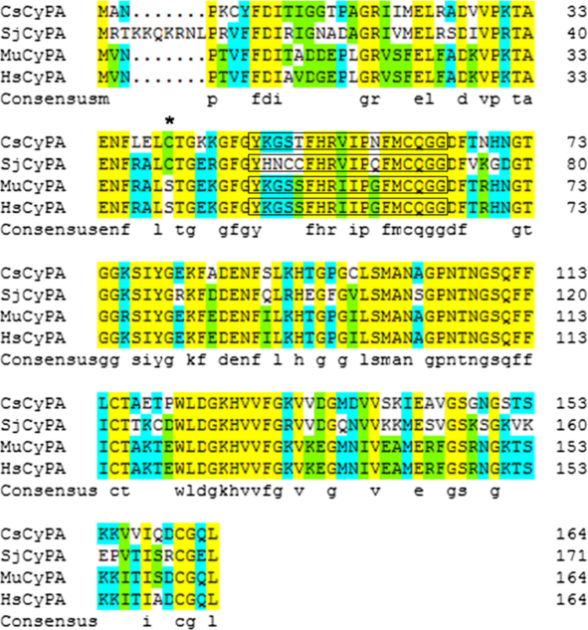
Alignment of CyPA Deduced Amino Acid Sequence from *C.sinensis* and Other Species. Letters with asterisk: site of forming disulfide bond. Letters with frame: PPIase catalytic area

The amino acid sequences of CsCyPA (AFI24615.1), SjCyPA (ACU78101.1), MuCyPA (NP_032933.1) and HsCyPA (AAI37059.1) were downloaded from Genebank (http://www.ncbi.nlm.nih.gov/nuccore/). The identities of amino acid sequence between CsCyPA and the other three were 66 %, 74 % and 74 % respectively. The putative PPIase catalytic area was identified by Motif Scan (http://myhits.isb-sib.ch/cgi-bin/motif_scan). The identities of amino acid sequence in catalytic area between CsCyPA and the other three were ordinal 92 %, 83 % and 83 % respectively. Sequence comparisons are shown in Fig 1.

### Immunoreactivity between rCyPA and anti-CsCyPAs

**Fig 2:**
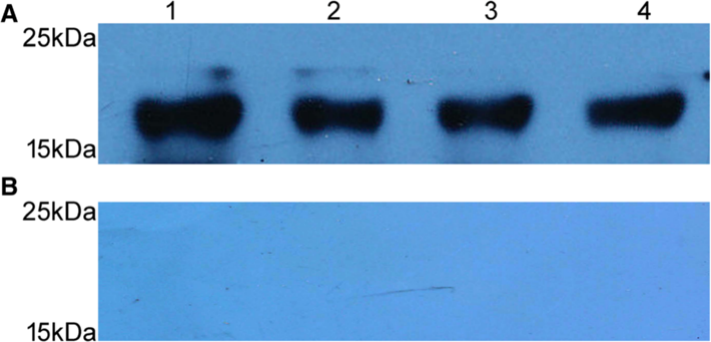
Western Blot Detected Immunoreactivity between Recombinant CyPA Protein and Anti-CsCyPAs. **a** (1, 2, 3, 4) rCsCyPA, rSjCyPA, rMusCyPA and rHsCyPA respectively probed with anti-CsCyPAs. **b** (1, 2, 3, 4) rCsCyPA, rSjCyPA, rMusCyPA and rHsCyPA respectively probed with PBS

Western blot analysis demonstrated that rCsCyPA, rSjCyPA, rMuCyPA and rHsCyPA could be recognized by anti-CsCyPAs, whilst not reacting with PBS in Fig 2. The results indicated that rCyPA of these species shared similar immunoreactivity with anti-CsCyPAs.

### Enzyme characteristics and inhibition experiments

**Fig 3:**
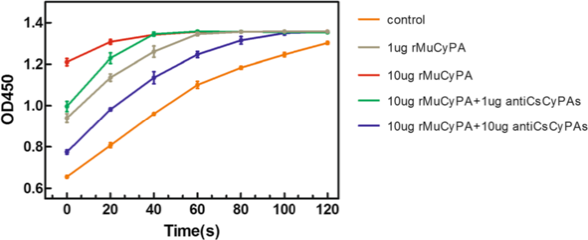
rMuCyPA Enzymatic Characteristics and Inhibition by Anti-CsCyPAs

Comparing 0 μg rMuCyPA reaction system (control group), 1 μg rMuCyPA reaction system and 10 μg rMuCyPA reaction system, rMuCyPA showed dose-dependent improvement on reaction rate in Fig 3. This result indicated that recombinant MuCyPA possesses PPIase activity. To investigate the ability of antibodies to inhibit enzymatic activity, we co-cultured 1 μg or 10 μg antibodies with 10 μg rMuCyPA for 1 h and then continued the reaction. Comparing 10 μg rMuCyPA reaction system, 10 μg rMuCyPA with 1 μg antibodies reaction system and 10 μg rMuCyPA with 10 μg antibodies reaction system, antibodies showed significant dose-dependent inhibition.

### Survival rates

**Fig 4:**
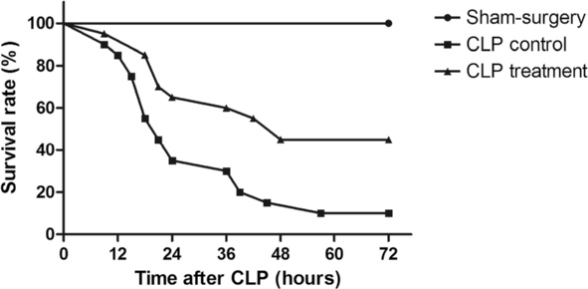
Survival Rates of Mice after CLP

The survival rates were analyzed 72 h after the CLP surgery. As is shown in Fig 4, the survival rate of Sham-surgery group was 100 %. Two of the twenty mice left in the CLP control 72 h subgroup, a survival rate of 10 %. Nine of the twenty mice left in CLP treatment 72 h subgroup, with a survival rate of 45 %. A statistically significant difference between the CLP control group and the CLP treatment group was observed (*p* < 0.05).

### CyPA level in serum

**Fig 5:**
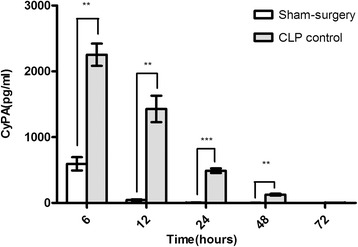
Detection of the Serum Level of CyPA in CLP and the Sham Group. *P < 0.05 versus sham-surgery group; **P < 0.01 versus sham-surgery group; ***P < 0.001 versus sham-surgery group

Serum samples were collected from both the sham-surgery group and the CLP control group. MuCyPA in the CLP control group was statistically higher compared with the sham group at 6, 12, 24 and 48 h (*p* < 0.05). There was no significant difference at 72 h (*p* > 0.05). In the CLP group, the CyPA level reached a climax at 6 h followed by a time-dependent decrease in Fig 5.

### Cytokine levels in serum

**Fig 6:**
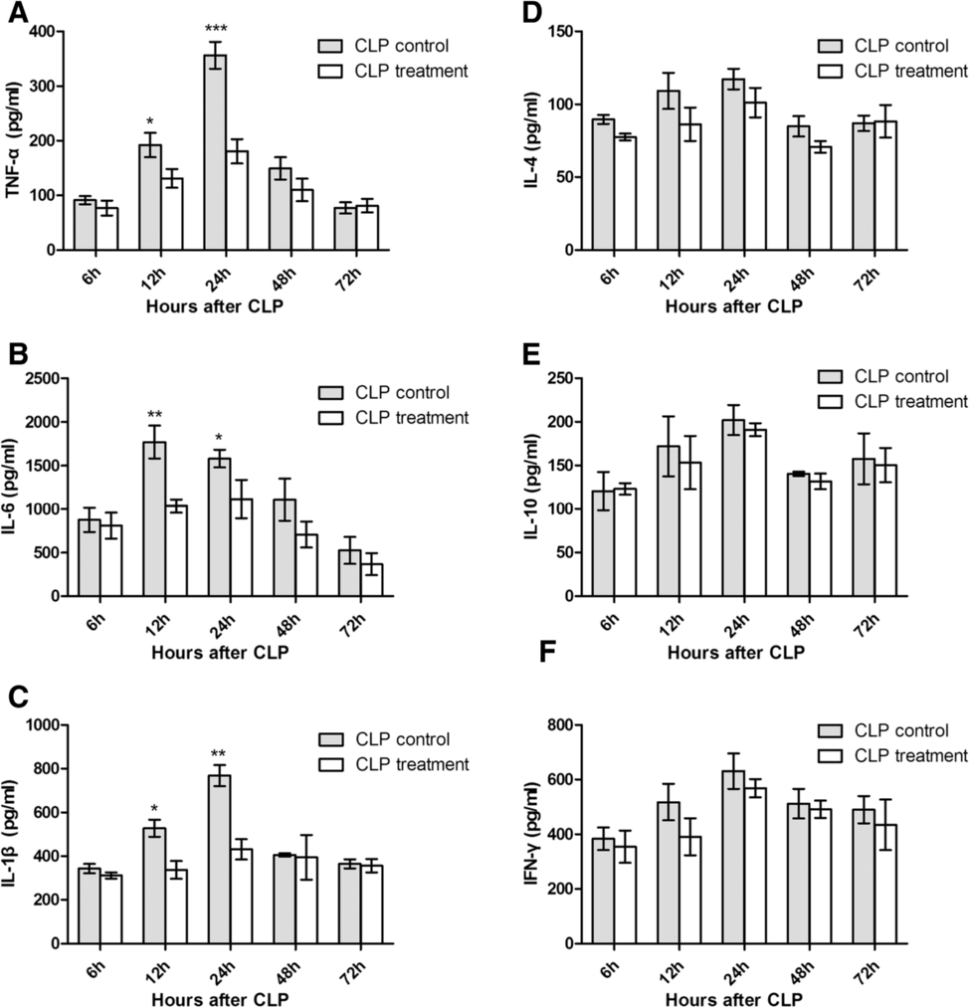
Change of Serum Cytokines in Mice after CLP. **a ** TNF-α concentration in mice serum; **b **IL-6 concentration in mice serum; **c **IL-1βconcentration in mice serum; **d **IL-4 concentration in mice serum; **e **IL-10 concentration in mice serum; **f** IFN-γ concentration in mice serum; *P < 0.05 versus CLP treatment group; **P < 0.01 versus CLP treatment group; ***P < 0.001 versus CLP treatment group

Cytokines which promoted inflammation like TNF-α, IL-6 and adjusted inflammation like IL-4, IL-10 and IFN-γ were chosen to represent the systematic inflammation level. Administration of antibodies significantly reduced the levels of TNF-α, IL-6 and IL-1β in serum compared with CLP control mice (Fig 6a, b, c) at 12 h and 24 h after CLP. Adjustment Cytokine had no statistical meaning (Fig 6d, e, f).

### Histology of lung and mesentery tissues

**Fig 7:**
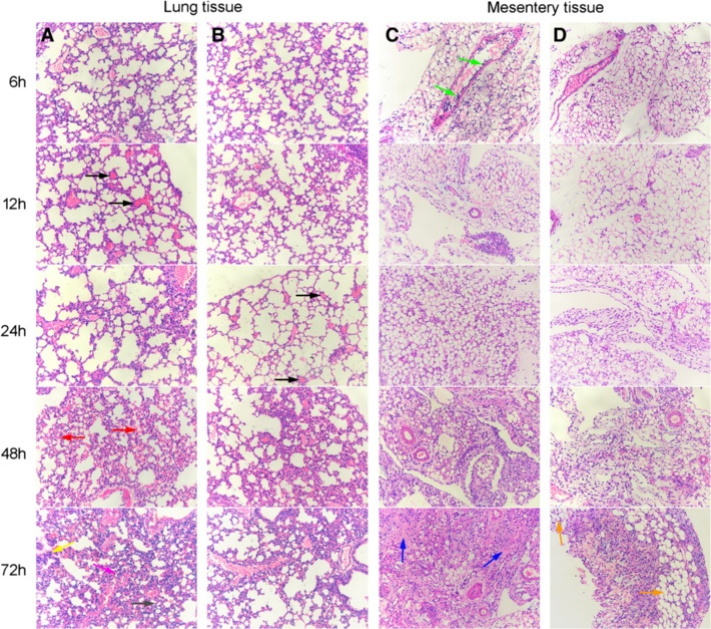
Histological Changes in Lung and Mesentery Tissues (hematoxylin-eosin; ×400). **a**, **c** lung and mesentery tissues of the CLP control group; **b**, **d** lung and mesentery tissues of the CLP treatment group. Black arrow: thrombus; Red arrow: hemorrhage; Pink arrow: alveolar consolidation; Yellow arrow: leukocyte aggregation; Gray arrow: alveolar wall thickening; Green arrow: inflammatory emigration; Blue arrow: mesentery fibrosis; Orange arrow: normal fat cells

H&E staining of the lung and mesentery of both the CLP control group and the CLP treatment group were shown in Fig 7. Lung tissue specimens in the CLP control group presented apparent thrombus at 12 h, and were characterized by leukocyte influx, edema, hemorrhage, wall thickening and alveolar consolidation at 48 h and 72 h (Fig.7a). In contrast, lung tissue in the CLP treatment group presented apparent thrombus at 24 h, and demonstrated leukocyte influx and edema without obvious hemorrhage and alveolar consolidation at 48 h and 72 h (Fig. 7b). Significantly, comparing lung tissue at 48 h and 72 h, pathological change seemed ameliorated in the CLP treatment group, while deteriorated in CLP control group.

Compared with the CLP treatment groups, the mesentery tissue in the CLP control group demonstrated more serious inflammatory reaction. Tons of inflammatory emigrated from vessel at 6 h. The boosting of cytolysis, fusiform cells and intercellular substance led to diffuse mesentery fibrosis at 72 h after surgery (Fig. 7c). In the CLP treatment group, inflammation was limited and some normal fat cells could still be observed at 72 h (Fig. 7d).

### Effects of antibodies on blood coagulation indicator

**Fig 8:**
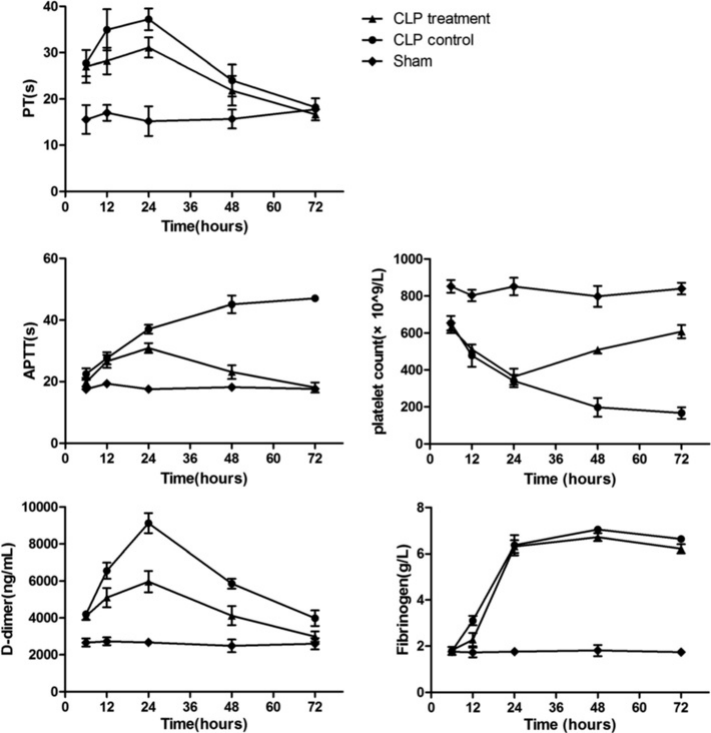
Blood Markers of DIC in CLP Treatment, CLP Control and the Sham Groups

As the laboratory diagnosis of diffuse intracellular coagulation (DIC) in the mice was defined by Minna JD [25], the CLP model successfully induced DIC, as the changes of blood markers were shown in Fig 8. Compared with the sham group, the platelet count, fibrinogen concentration, PT, aPTT and D-dimer in the CLP control group began to change significantly at 6 h post-CLP.

Comparing the CLP control group with the CLP treatment group, there were significant differences on the indicators including PT, aPTT, D-dimer and platelet count. Furthermore, antibodies in mice obviously ameliorated these four indicators at 48 h and at 72 h after the surgery. Although there was no significant difference in fibrinogen concentration, the significant changes of other indicators showed strong evidences for the efficacy.

### Viability of ECs with MuCyPA and anti-CsCyPAs by CCK-8 assays

**Fig 9:**
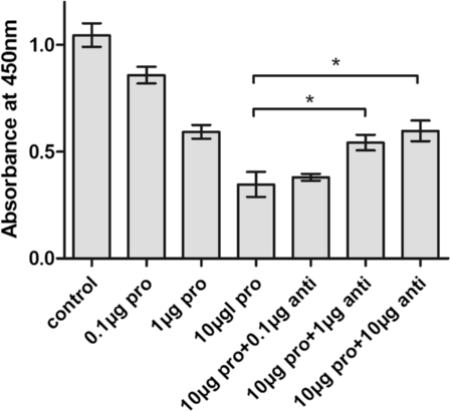
Detection of the Viability of EC with rMuCyPA and Anti-CsCyPAs. X axis final supernatant content of rMuCyPA and anti-CsCyPAs in each speed; Y axis presented the absorbance at OD_450nm_ in each well after incubated with 10 μl of CCK-8 solution at 37 °C for 2 h. Pro is the abbreviation of rMuCyPA; anti is the abbreviation of anti-CsCyPAs; *P < 0.05

To evaluate the viability of ECs after being co-cultured with rMuCyPA, ECs were incubated in 0 μg (control), 0.1 μg, 1 μg and 10 μg MuCyPA for 24 h, and cell viability was measured using the CCK-8 assays in Fig 9. Compared with the control group, the viability of ECs exposed to rMuCyPA showed a dose-dependent decrease. In the inhibition test, 0.1 μg, 1 μg and 10 μg antibodies were co-cultured with 10 μg MuCyPA for 1 h before reaction. Compared with 10 μg MuCyPA group, the result showed significant increase on the percentage of cells in the 1 μg and 10 μg antibody groups in a dose-dependent manner (*p* < 0.05). There was no significant difference between 10 μg MuCyPA group and 0.1 μg antibodies group (*p* > 0.05).

## Discussion

In this study, we found that the blood level of MuCyPA increased in the development process of sepsis induced by CLP surgery. Anti-CsCyPAs approach had a beneficial effect on the survival rate of CLP mice and obviously improved the observational indicators including blood coagulation, lung and mesentery pathology and cytokine levels in serum.

In 1992, Barbara et al. [9], found that lipopolysaccharide-activated macrophages could secrete CyPA as a proinflammatory factor, the role of which in the inflammatory disease was evaluated in following studies. It has been found that CyPA significantly increased in the development process of infection in a couple of studies. For example, in 2007, Dear et al., found that CyPA highly increased in the liver after CLP [26]. Huang et al., reported that CyPA expression was modulated in peripheral lymphocytes from *Pseudomonas aeruginosa* induced sepsis [27] and *Staphylococcus aureus* invasion [28]. Similarly, in our study, the MuCyPA in serum, which might originate from the cells receiving inflammatory stimuli increased in abundance and reached the climax at 6 h in Fig 5.

The mechanism of CyPA working as a proinflammatory factor unequivocally depended on the combination with CD147 receptor [11]. CD147, a single transmembrane glycoprotein, is widely expressed on the cell surface which can be expressed in all of the white blood cells, platelets and endothelial cells in most normal tissues in weak or no expression [29]. The proline 180 and glycine 181 residues in the extracellular domain of CD147 were critical for signaling and chemotactic activities mediated by CD147 which could be accomplished by the PPIase activity of CyPA [30]. The activated CD147 could transfer information into cells, leading to chemotaxis, release of factor and apoptosis of ECs which all induced deterioration of the sepsis. Significantly, the expression of CD147 in membrane was obviously increased in sepsis as was CyPA. Furthermore, in 1997, Tegeder et al. reported that CyPA PPIase activity was significantly higher in patients with severe sepsis compared with healthy subjects. In addition, elevated PPIase activity was associated with high mortality [31].

In this study, anti-CsCyPAs could recognize and cross-react with MuCyPA and effectively inhibit the PPIase enzymic activity as the obvious identity of CyPA amino acid sequence in different species, which may in turn induce inhibition on the activity of CD147. Comparing the CLP control group and the CLP treatment group in Figs 6 and 7, antibodies approach had a significantly favorable effect on the improvement of inflammation. The proinflammatory cytokines including IL-6, IL-1β and TNF-α were obviously lower compared with the control group, while anti-inflammatory cytokines including IL-10, IL-4 and IFN-γ presented no significant difference between the two groups. The level of CyPA increased at an early stage and followed by a drop to the normal in 72 h, which suggested that CyPA plays a primary role in the SIRS stage of sepsis, but is not significantly involved with the compensatory anti-inflammatory response syndrome (CARS) stage of sepsis. This reaction might alleviate the systematic inflammatory reaction and reduce the possibility of SIRS. Meanwhile, CyPA also serves as a key determinant for TNF-α inducing ECs apoptosis, which could also increase vascular permeability and induce hypovolemic shock [11]. Therefore, the protection on ECs might be the main reason behind the increased survival rate in the early stage. DIC is a common complication of sepsis, and is associated with a poor prognosis. The blood coagulation indicator in the treatment group also showed a favorable progress after 24 h. Finally, the immediate outcome of survival rate adequately proved the efficacy of anti-CsCyPAs on sepsis.

There were three limitations of this study that warrant notice. Firstly, the efficacy of antibodies might correlate with the combination of antigen and antibodies that inhibit CyPA from combining with CD147. This mechanism might play an important role as inhibition of enzyme. Secondly, no *in vivo* experiment on the effect of antibodies was carried out. It was found to be impossible to separate pure mouse peritoneal macrophage due to the open wound and persistent infection in abdomen after reviewing many studies. Finally, anti-CyPA antibodies had been verified to increase in some autoimmune diseases like rheumatoid arthritis, systemic lupus, erythematosus and so on, thus the safety of this “parasite medication” should be further verified in further studies.

## Conclusion

Anti-CsCyPA antibodies had a verified beneficial effect on sepsis induced by CLP surgery by inhibiting the PPIase activity. The indicators of pathology, cytokines, blood coagulation indicator and survival rate all generally improved. As far as its safety is concerned, the preventative injection of CsCyPA might be controversial, but anti-CsCyPAs as an alternative treating approach for acute sepsis patients may be considerable.
